# Lumbar Spine Surgery in a Patient With Recent Monkey Bite Receiving Rabies Immunoglobulin: A Case Report on Perioperative Anesthetic Challenges

**DOI:** 10.7759/cureus.111066

**Published:** 2026-06-17

**Authors:** Vivek Shankar, Ira Pachori, Pranav Kapoor

**Affiliations:** 1 Orthopedics, All India Institute of Medical Sciences, New Delhi, IND; 2 Orthopedic Surgery, All India Institute of Medical Sciences, New Delhi, IND

**Keywords:** case report, orthopedic spinal surgery complications, prolapsed intervertebral disc, rabies postexposure prophylaxis, transforaminal lumbar interbody fusion (tlif)

## Abstract

Rabies is a universally fatal acute zoonotic disease caused by rhabdovirus, and postexposure prophylaxis (PEP) remains the mainstay of prevention. Patients who sustain animal bites may require urgent or elective surgical interventions for unrelated conditions while simultaneously undergoing rabies prophylaxis. The perioperative team often faces uncertainty regarding the safety of anesthesia and surgery in patients receiving rabies vaccines or rabies immunoglobulin (RIG). This report discusses such a scenario in a patient posted for elective lumbar spine surgery.

We report the case of a male in his mid-60s with chronic low back pain and bilateral lower limb radiculopathy, with the left side more involved, diagnosed with a prolapsed intervertebral disc at the L5-S1 level with lumbar canal stenosis. Following failed conservative management for six weeks, he was planned for decompression and transforaminal lumbar interbody fusion (TLIF) at the L5-S1 level. On the day before surgery, he received a scratch from a monkey, classified as a Grade III wound, which was managed, and PEP was initiated, including human RIG and a four-dose intradermal vaccine regimen. Surgery was deferred till completion of PEP, following which he underwent TLIF at the L5-S1 level. There were no intraoperative adverse events. The immediate postoperative course was uneventful, with significant symptomatic improvement. The surgical site was found to be healthy on postoperative day 2, and the patient was discharged on the next postoperative day.

Our case shows that timely administration of rabies PEP in the form of RIG and vaccine, along with postponement of elective surgery, does not preclude the patient from safely undergoing elective spine surgery in the future, provided vaccination schedules are not interrupted and adequate time is allowed after immunoglobulin administration. In our patient, lumbar TLIF was performed after completion of the PEP schedule, and he had an uneventful intraoperative and postoperative course, with a good clinical recovery. This highlights the importance of appropriate coordination between infectious disease management and individualized surgical planning, along with careful perioperative and postoperative monitoring, to balance the urgency of spinal disease and the need for effective rabies prevention for optimal surgical outcomes.

## Introduction

Rabies is a lethal and highly prevalent infectious disease, with an incubation period of usually two to three months, which rarely exceeds one year. It has a 100% fatality rate once symptoms set in, manifesting as endemic fear, water phobia, pharyngeal muscle spasm, and progressive paralysis. Despite being entirely preventable through timely postexposure prophylaxis (PEP), it causes tens of thousands of deaths annually, predominantly in low- and middle-income countries [1]. Management is performed according to the 2014 WHO guidelines [2], which consist of prompt wound washing and administration of rabies immunoglobulin (RIG) and rabies vaccine, using any of the described regimens. Our institute follows the four-dose intradermal Updated Thai Red Cross regimen [3].

While rabies PEP is widely used, guidance regarding the perioperative management of patients who need surgery while actively receiving PEP is sparse. Understanding of the interaction between surgery, anesthesia, and vaccine-induced immunity remains poor. It is hypothesized that surgical stress may induce transient immunosuppression via hypothalamo-pituitary-adrenal axis activation and increased cortisol secretion, raising questions about perioperative vaccine efficacy. Certain anesthetic agents, such as ketamine, have been implicated in isolated reports of PEP failure, while neuraxial anesthesia may create a challenge if paralytic rabies develops during the postvaccination period and neuro-deficits are mistakenly attributed to regional block. Although emergency surgeries such as cesarean section under general anesthesia and orthopedic surgery under neuraxial anesthesia have been reported safely in patients receiving rabies PEP, this evidence is limited to isolated case reports. Hence, there is an uncertainty when balancing the need for timely surgical intervention against the theoretical risk of compromising vaccine effectiveness or delaying the diagnosis of rabies after vaccination. Further, there are no consensus guidelines specifically addressing the optimal timing of elective surgery or the choice of anesthetic technique and agents.

We report the case of a patient who required orthopedic surgery while on rabies PEP, contributing to the limited literature on perioperative considerations, anesthetic decision-making, and successful clinical outcomes.

## Case presentation

A male in his mid-60s presented with chronic low back pain for 10 years, bilateral lower limb radiculopathy involving the left side more than the right for 6 years, and paresthesia with tingling. His walking distance was limited to <50 m. There was no history of weakness, bowel/bladder involvement, or trauma. There was a history of pulmonary tuberculosis in early childhood, for which he took antitubercular therapy for an unknown duration. He was hypertensive on rosuvastatin, clopidogrel, and cilnidipine; diabetic on dapagliflozin, sitagliptin, metformin, and glimepiride; and hypothyroid on oral thyroxine. He had a hemorrhoid excision in 2019.

Local examination revealed lumbar tenderness (L5-S1), loss of lumbar lordosis, and restricted straight leg raise (left: 80°, right: 70°). Neurological examination showed no deficits. Preoperative X-ray imaging was performed (Figure 1). MRI lumbar spine with whole spine screening revealed multilevel degenerative disc disease at L2-L3, L3-L4, and L4-L5 levels with a prolapsed intervertebral disc at the L5-S1 level with lumbar canal stenosis (Figure 2). Following six weeks of failed conservative therapy with analgesics and spinal physiotherapy, decompression transforaminal lumbar interbody fusion (TLIF) at the L5-S1 level was planned. As per preanesthetic protocol, clopidogrel was discontinued five days and dapagliflozin three days before scheduled surgery. Morning oral hypoglycemic agent doses were omitted, and the patient was managed with s/c insulin on a sliding scale. A test dose of cefuroxime was given.

**Figure 1:**
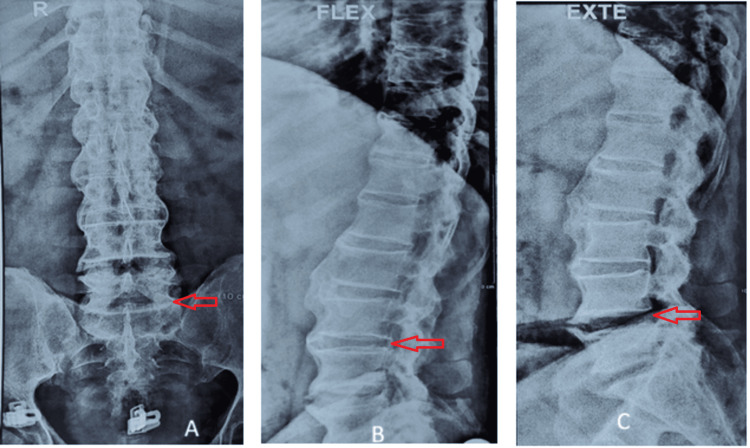
Preoperative X-ray lumbar spine: (A) anteroposterior, (B) lateral flexion, and (C) lateral extension

**Figure 2:**
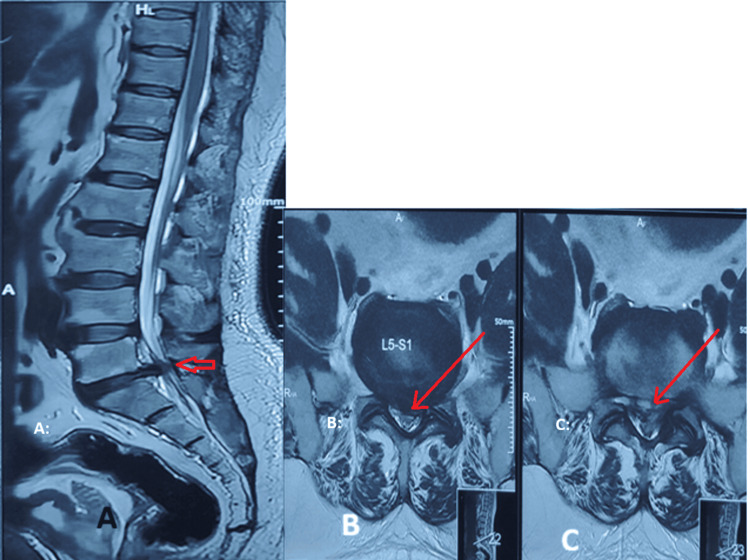
Preoperative MRI lumbar spine: (A) sagittal sections showing PIVD L5-S1 and (B, C) axial sections of L5-S1 space showing lumbar canal stenosis PIVD, prolapsed intervertebral disc.

On the day before surgery, the patient received a scratch from a monkey (Grade III) [4] on the right flank region (Figure 3). The patient was immediately taken to emergency, and PEP was initiated. The wound was flushed with water for 15 minutes, followed by disinfection with tincture iodine. 20 IU/kg of human RIG was infiltrated around and into the wound as much as anatomically possible, and the rest was intramuscularly injected into the right anterior thigh. A four-dose vaccine regimen was implemented as per WHO guidelines, and the zeroth dose of 0.1 mL intradermal vaccine was given, along with 0.5 mL intramuscular injection of tetanus toxoid.

**Figure 3:**
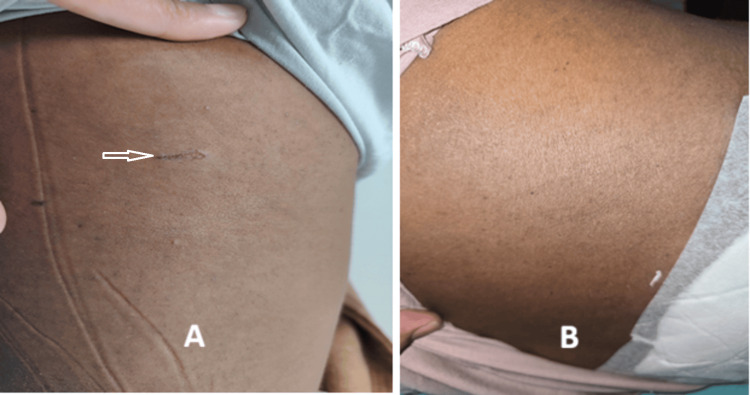
(A) Monkey scratch wound on the patient’s right flank and (B) healed scratch wound after 28 days

Elective lumbar spinal surgery was deferred while the regimen was completed, and the patient received the vaccine on days 3, 7, and 28. The site scratch was regularly evaluated. The patient was planned for surgery after completion of the PEP course. At the time of the preanesthetic checkup, the patient was afebrile and vitally stable, with a healed scratch wound, no signs of infection, and no neurological deficits related to the bite. Systemic examination was unremarkable apart from the spine disease. Baseline hematological and biochemical investigations were within normal limits, and there were no contraindications to anesthesia.

TLIF L5-S1 was performed one month after the patient received the monkey bite, a week after completion of the PEP course. The intraoperative course was uneventful. The surgery lasted for three hours with a blood loss of approximately 250 mL, managed with crystalloids and colloids. Postoperative recovery was uneventful, with significant symptomatic improvement and a healthy surgical site. Patient was mobilized with a lumbar spine corset in situ on postoperative day 1. Wound inspection was performed on postoperative day 2 and found to be healthy. The patient was discharged on postoperative day 3 and followed up in routine OPD. Suture removal was performed on postoperative day 15 after wound examination. Patient remained symptom-free and remained well at 3, 6, and 12 months follow-up (Figures 4, 5).

**Figure 4:**
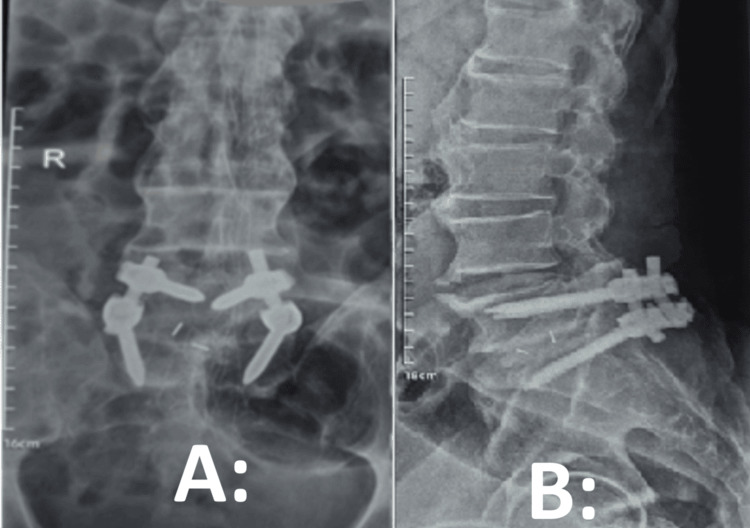
Postoperative X-ray lumbar spine: (A) anteroposterior and (B) lateral view showing TLIF L5-S1 taken on postoperative day 1 TLIF, transforaminal lumbar interbody fusion.

**Figure 5:**
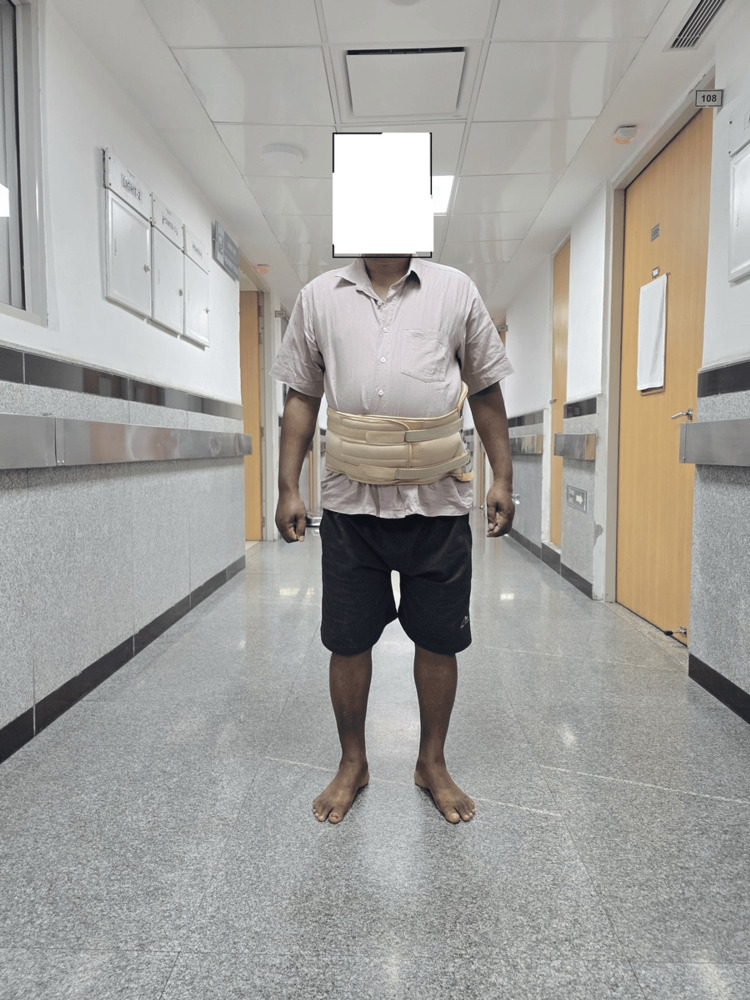
Patient remained symptom-free and well at the third month postoperative

## Discussion

Rabies is a preventable viral, zoonotic, neglected tropical disease that infects mammals, including dogs, cats, livestock, and wildlife, and spreads via saliva, usually through bites, scratches, or direct mucosal contact. It affects the central nervous system and is virtually 100% fatal once clinical symptoms appear.

Prompt PEP consisting of thorough wound washing and administration of a course of human rabies vaccine and, when indicated, RIG can prevent rabies deaths.

There is limited literature on the safety of anesthesia and surgery in patients undergoing elective surgery who are receiving antirabies vaccination. A case report by Fescharek et al. [5], which studied 15 cases of PEP failure, concluded that patients with additional risk factors may pose higher chances of PEP failure, with immunosuppression identified as a risk factor. An article by Cusack et al. [6] elaborates on the immunosuppressive effects of major surgery due to immediate postoperative increase in ultradian pulses in ACTH, along with a persistent elevation of plasma cortisol in the postoperative period.

While immunosuppression caused by commonly used anesthetic agents and techniques may lead to decreased vaccine effectiveness or an increased risk of complications, there is no direct evidence of the same in the existing literature. Emergency surgeries have been performed safely on patients while receiving PEP. Ghodki et al. [7] reported a 19-year-old primigravida who received two rabies vaccine doses and then underwent emergency C-section under general anesthesia, completing the PEP in the postoperative period with an uneventful course. Based on consensus produced by an international survey conducted among members of various pediatric anesthetics by Short et al. [8], it is recommended that elective surgery and anesthesia should be postponed for one week after inactivated vaccination and three weeks after live-attenuated vaccination. Although evidence remains sparse, anesthetic choice could influence outcomes, as shown by the case report by Fescharek et al. [5], which attributed one case of pediatric PEP failure due to ketamine being used as an anesthetic agent. Theoretically, regional anesthesia allows for more attenuation of the systemic stress response and better preservation of immune function, as stated in a review article by Horlocker et al. [9] and a case study by Rewari et al. [10] on a patient receiving PEP who was administered neuraxial (spinal) anesthesia for emergency orthopedic surgery, which even documents a successful recovery for the patient. Conversely, use of neuraxial anesthesia in a patient on rabies PEP could pose a diagnostic dilemma if the presentation is paralytic without the typical excitation and hydrophobia, where the neurological deficit could be mistakenly attributed to the regional block, as was presented in a case report by Chakravarty et al. [11].

Although our case has a relatively short follow-up period to judge spinal fusion outcomes, of one year, it highlights the clinical quandary faced by anesthetists and surgeons when a patient on antirabies vaccine requires surgical intervention, as its need in the setting of the patient having received rabies vaccination has to be balanced with possible morbidity or disability that could occur by deferring the procedure. The key principle is not to interrupt or delay the vaccine schedule. Caution is required in avoiding perioperative immunosuppressive medications unless absolutely essential. Emphasis is also needed on a clearer postoperative neurology assessment if neuraxial techniques are considered. While this case report does demonstrate successful management of one patient, a larger sample size with longer follow-up is needed to establish recommendations for broader clinical practice.

## Conclusions

Though elective surgery during active rabies PEP is not absolutely contraindicated, existing studies favor delaying elective surgeries, lumbar spine surgery in our case, until at least one week after inactivated vaccine doses to mitigate the theoretical risk of reduction of vaccine efficacy from immune interference from surgical stress or anesthesia.

In case of an absolutely necessary procedure, choice of anesthetic agent and/or technique needs to be individualized, while considering the possible consequences of general anesthesia and neuraxial anesthesia, with meticulous continuation of the PEP schedule and thorough documentation of neurological deficit, if any, in the preoperative and postoperative periods, along with avoidance of perioperative immunosuppressive therapy.
